# The Future Potential of Biosensors to Investigate the Gut-Brain Axis

**DOI:** 10.3389/fbioe.2021.826479

**Published:** 2022-01-14

**Authors:** Jiefei Wang, W. Seth Childers

**Affiliations:** Department of Chemistry, University of Pittsburgh, Pittsburgh, PA, United States

**Keywords:** depression, gut-brain axis, biosensors, metabolites, synthetic biology

## Abstract

The multifaceted and heterogeneous nature of depression presents challenges in pinpointing treatments. Among these contributions are the interconnections between the gut microbiome and neurological function termed the gut-brain axis. A diverse range of microbiome-produced metabolites interact with host signaling and metabolic pathways through this gut-brain axis relationship. Therefore, biosensor detection of gut metabolites offers the potential to quantify the microbiome’s contributions to depression. Herein we review synthetic biology strategies to detect signals that indicate gut-brain axis dysregulation that may contribute to depression. We also highlight future challenges in developing living diagnostics of microbiome conditions influencing depression.

## Introduction

**FIGURE 1:**
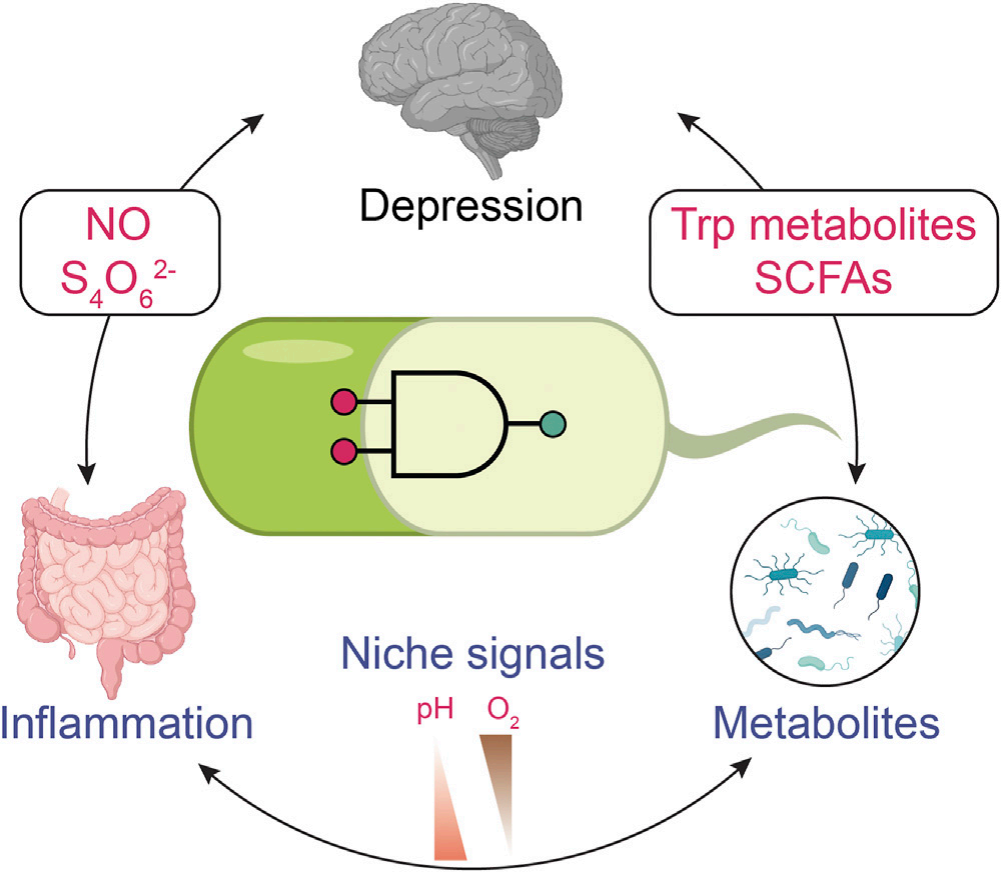
Monitoring depression with biosensors through the gut-brain axis. Engineered biosensors might be used for monitoring alterations in gut metabolism that could impact depression. Gut inflammation plays a role in mental disorders. Nitric oxide (NO) and tetrathionate (S_4_O_6_^2-^) present inflammation biomarkers that the engineered bacterial sensor can detect. Gut microbiota can alter the metabolites. For example, tryptophan metabolites and short-chain fatty acids (SCFAs) are associated with human behavior problems. The signals indicating mental disease can be coupled with general signals in the digestion system, such as pH and O_2_ level. The specificity of biosensors can be improved through multi-input logic gates. The icons of the brain, gut, and microbes are adapted from ‘Brain (lateral view)’ “Intestines” and “Spirillum (flagella)” by BioRender.com (2021). Retrieved from https://app.biorender.com/biorender-templates.

The bidirectional nature of the gut-brain axis links the gut microbiota with psychological behaviors (Mayer, 2011; Cryan and Dinan, 2012). Within this gut-brain axis, metabolites produced by the microbiota alter the host immune, metabolic and neural pathways that contribute to behavioral changes (Zheng et al., 2016). The interconnections between gut microbiota metabolites and host behavior are evidenced by human fecal transplantation experiments into mice (De Palma et al., 2017). These studies suggest that microbiota composition leads to dysregulation of the gut-brain axis, which has been proposed to influence depression (Pinto-Sanchez et al., 2017; Kelly et al., 2019). Several in-depth reviews have examined the rich interconnections with the gut-brain axis (Rutsch et al., 2020; Margolis et al., 2021; Morais et al., 2021). Here we introduce connections between the microbiome and mental health that present opportunities for synthetic biology (Figure 1).

A current diagnostic test to quantify biomarkers may involve samples from cerebral spinal fluid and blood analyzed by techniques such as high-performance liquid chromatography (HPLC), mass spectrometry (MS), and nuclear magnetic resonance (NMR) (Haroon et al., 2020; Du et al., 2021; Pu et al., 2021). In contrast, synthetic biology offers next-generation diagnostic strategies through the engineering of probiotic bacteria. Early successes of the synthetic biology approaches are in clinical trials targeting metabolic diseases and cancers (McNerney et al., 2021). These strategies have been reviewed (Song et al., 2019; Barra et al., 2020; Lazar and Tabor, 2021; McNerney et al., 2021), including the engineering of probiotics to produce synthetic metabolites and therapeutic proteins (Kang et al., 2020). Collectively, these technologies have enabled the sensing of tumors and the release of therapeutics (Chien et al., 2017).

We are now poised to leverage these new synthetic biology tools to study, diagnose, and treat mental health disorders. Indeed, psychobiotics, including *Bifidobacterium* and *Lactobacillus* families (Mayer et al., 2014) have been studied to positively influence the gut-brain axis (Sarkar et al., 2016), and exhibited improved mental health (Dinan et al., 2013). Altered gut microbiota profiling was detected in patients, and probiotic treatments were shown to have promising improvement for depression (Kelly et al., 2019; Flux and Lowry, 2020). As a chassis for biosensing technologies, both bacteria and bacteriophages present opportunities to characterize the gut-brain axis.

On the one hand, bacteriophages can be engineered as biosensors to target specific bacteria within the microbiota (Bhardwaj et al., 2017). With specificity towards selected bacterial species, bacteriophages have been proposed as therapeutic vectors (Sulakvelidze et al., 2001; Summers, 2001), and technologies using bacteriophages are being evaluated in clinical trials (Lu and Koeris, 2011; Lenneman et al., 2021). Notably, protein engineering strategies upon the T3 phage tail fiber protein to engineer host-range specificity (Yehl et al., 2019). Bacteriophages thus present significant opportunities to track specific bacterial species that have been associated with mental health.

In a complementary manner, bacteria can be engineered as biosensors that detect the collective metabolic output from microbes and hosts. Emerging evidence of the gut-brain axis suggests a qualitative correlation between metabolite levels in the gut and the brain. With further development of technologies, biosensor surveillance of gut metabolites may lead to non-invasive, precise, personalized medicine for mental health-associated diseases (Zmora et al., 2018). Moreover, biosensors have the potential to quantitively score conditions within the gut that contribute to mental health. Sensing microbe species and metabolites collectively might help distinguish primary contributors to the changes in metabolite levels. However, the biosensor applications will be challenged by the specificity of metabolite signals, the dynamic range of signals, promoter leakiness, and potential crosstalk with other pathways. This minireview focuses on the current state and future challenges of bacterial biosensors that can monitor the impact of the microbiota upon the gut-brain axis.

## Biosensors for the Detection of Gut-Brain Axis Dysregulation

### Tryptophan Metabolites as Potential Biomarkers of Depression

Tryptophan is metabolized into three downstream pathways: 5-hydroxytryptamine, kynurenine, and indole pathways (Hubbard et al., 2015; Agus et al., 2018; Gheorghe et al., 2019; Kaur et al., 2019; Benech et al., 2021; Modoux et al., 2021). Variations in how the gut microbiota metabolizes tryptophan might play a role in depression (Kelly et al., 2016). Indole functions as an interkingdom signaling molecule (Lee et al., 2015) through stimulating the aryl hydrocarbon receptor (AhR) pathway (Agus et al., 2018; Liu et al., 2021). Metagenomic analysis of enzymes that produce indole (i.e., TnaA) suggests that gut microbiota from different individuals has a varied capacity to synthesize indole, leading to variations in indole within humans (Jaglin et al., 2018). Other studies in mice under chronic mild stress observed that indole production correlated with changes in mice behavior (Mir et al., 2020), while overproduction of indole-induced anxiety-like behavior in rats (Jaglin et al., 2018). In contrast, studies in mice have shown that indole stimulation of the AhR regulates neurogenesis in the adult hippocampus, a critical step in recovering from depression (Wei et al., 2021). Thus, maintenance of indole levels appears critical for proper gut-brain axis regulation (Jaglin et al., 2018; Mir et al., 2020).

### Biosensors for Indole Metabolites

Protein engineering of the tryptophan repressor protein could enable rapid detection of tryptophan-derived metabolites. A fluorescence resonance energy transfer (FRET) sensor for the signaling molecule indole-3-acetic acid (I3AA) was reported with such a protein engineering approach. The sensor was designed by altering the specificity of the *E. coli* tryptophan repressor for I3AA binding (Herud-Sikimić et al., 2021). The I3AA FRET sensor was tested for tracking dynamic fluctuations of I3AA signals in plants. The same strategy can be applied to engineer FRET biosensors for other indole metabolites. Moreover, future preclinical studies applying similar FRET biosensors may enable three-dimension mapping of indole metabolism along the digestive tract.

**FIGURE 2:**
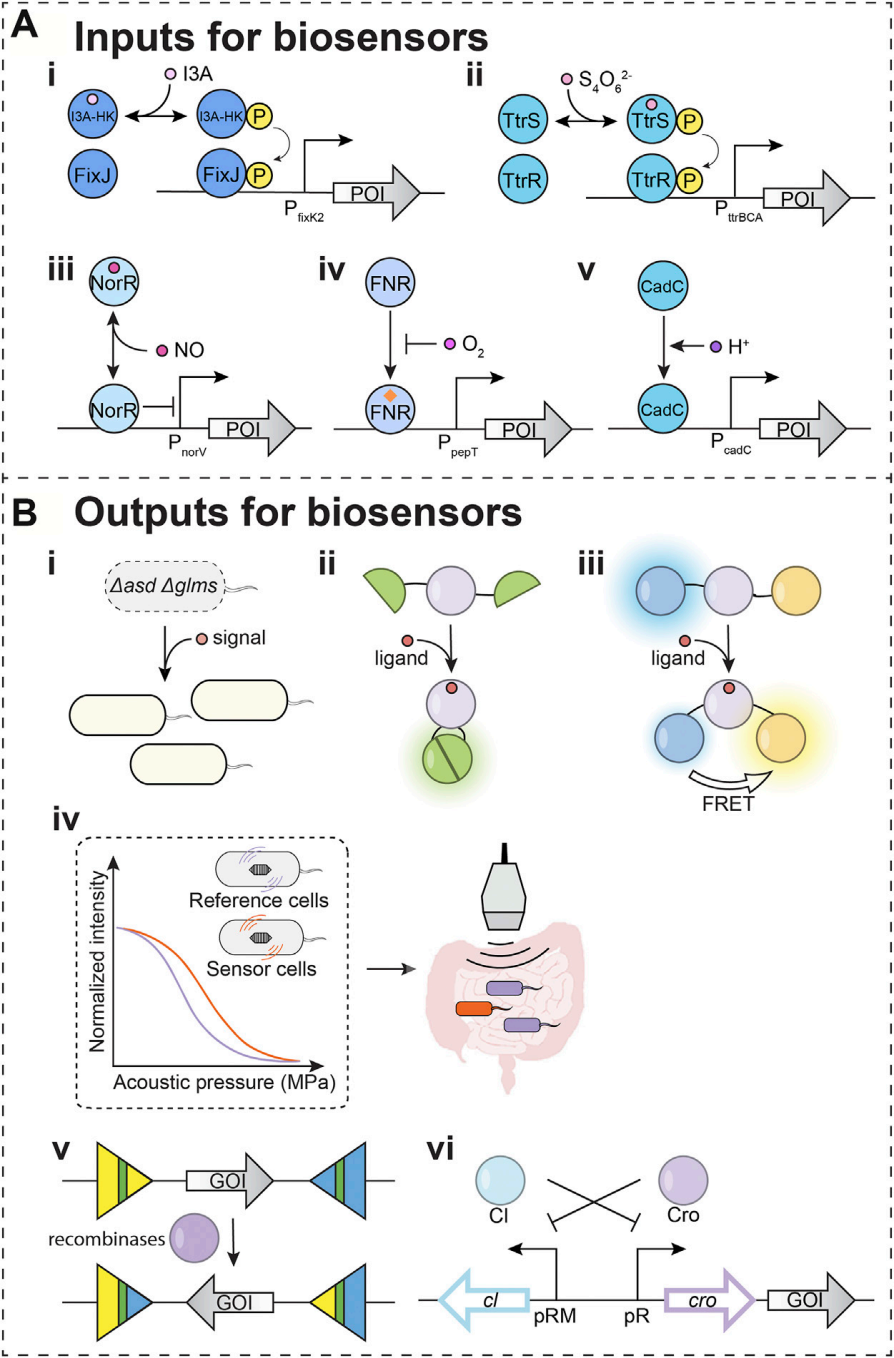
Input and output modules for biosensors. **(A)** Input modules for detecting potential depression-associated signals. POI: protein of interest. (**i–ii**) Two-component systems (TCSs) that were used in biosensors. (**i**) I3A-HK/FixJ: an engineered sensor for indole-3-aldehyde (I3A). (**ii**) TtrSR: tetrathionate sensor (**iii–v**) Transcription factors and sigma factors that have been used as biosensor parts. (**iii**) NorR: nitric oxide sensing regulator. (**iv**) FNR: fumarate and nitrate reduction regulatory protein. (**v**) CadC: a membrane-bound transcriptional regulator. **(B)** Output modules for biosensors (**i–iv**). Outputs for *in situ* detection. (**i**) Essential genes are used as outputs. *asd*: aspartate-semialdehyde dehydrogenase; *glms*: glucosamine-6-phosphate synthase. Both *asd* and *glms* encoded proteins are involved in Gram-negative bacterial cell wall biosynthesis. In the engineered *S. Typhimurium* ELH1301 *Δasd Δglms* strain, *asd* and *glms* can be induced once sensing the signals. The compensation of the essential genes will increase the cell population in the designated environment. (**ii**) Split fluorescent protein is used as output for the signal. The ligand binding to the sensing domain induces a conformational change, which leads to the reunion of domains from a split fluorescent protein. The reunited fluorescent protein generates detectable signals. (**iii**) The fluorescence resonance energy transfer (FRET) is used for output detection. The ligand binding to the sensing domain induces a conformational change, which leads to the FRET for detectable output fluorescent signal. (**iv**) The operon for gas vesicles can serve as the output for a biosensor. The expressed proteins can assemble into gas vesicles, allowing ultrasound signals from tissue in depth. (**v,vi**) Outputs for delayed detection. (**v**) Recombinases can irreversibly invert gene of interest (GOI) on the DNA upon detecting signals. The readout can be measured through DNA sequencing for complex circuits. (**vi**) The toggle circuit controls the GOI as a memory module. The icon of the gut is adapted from “Intestines” by BioRender.com (2021). Retrieved from https://app.biorender.com/biorender-templates.

Other than indole, indole-3-aldehyde (I3A) may also activate the AhR receptor and regulate central nervous system inflammation (Rothhammer et al., 2016). To detect I3A, a biosensor was engineered based on signaling parts from a bacterial two-component system (TCS) (Figure 2Ai). The engineered histidine kinase is a hybrid protein carrying an I3A-sensing Per-Arnt-Sim (PAS) domain (Wang et al., 2021). The optimization within the promotor region improved the dynamic range and resulted in ∼35 fold change in mCherry signal upon sensing I3A. The engineered *E. coli* selectively sense I3A over other indole metabolites. However, at higher concentrations, the I3A sensor can respond to indole and I3AA. This I3A-sensing biosensor raises the potential of developing biosensors to investigate the impact of indole metabolites on mental health disorders.

### Gut Inflammation and Its Connections to Depression

The microbiota may also stimulate gut inflammation (Miller and Raison, 2016), which plays a role in depression (Liu et al., 2020). The immune system signals to the brain through inflammatory cytokines (Flux and Lowry, 2020). Recent research found that inflammation can increase the permeability of the blood-brain barrier (Varatharaj and Galea, 2017) to allow cytokines to cross. At the same time, microbes can modulate gut-associated lymphoid tissue, resulting in various cytokines (Desbonnet et al., 2010; Rudzki and Szulc, 2018). Therefore, quantification of inflammation may assist in developing treatment regimens for depression.

### Tetrathionate Biosensors for Inflammation

Tetrathionate is a small molecule signal indicating inflammation (Winter et al., 2010). Host cells convert the toxic hydrogen sulfide generated from bacteria to the thiosulphate (Levitt et al., 1999), which interacts with reactive oxygen species generated during gut inflammation to form the tetrathionate (Winter et al., 2010). *Salmonella typhimurium* sense the tetrathionate by TCS TtrSR, which regulates tetrathionate reductase (TtrBCA) in the tetrathionate respiration (Hensel et al., 1999). The *E. coli* Nissle 1917 was successfully engineered to carry a TtrSR for detecting tetrathionate in the inflamed mice gut (Figure 2Aii) (Daeffler et al., 2017). The Silver lab further applied this tetrathionate sensing system to regulate a memory toggle switch within the mouse gut commensal *E. coli* NGF-1 strain. Their studies demonstrated that the bacterial sensor with the memory circuit maintained and recorded tetrathionate signals after 160 days in mice gut (Riglar et al., 2017).

### Nitric Oxide Biosensors for Inflammation

The nitric oxide (NO) also serves as an inflammatory biomarker (Kimura et al., 1997; Sharma et al., 2007). NO is a water-soluble gaseous small molecule synthesized from *l* -arginine by nitric oxide synthases (NOSs) in mammalian cells. The expression of inducible nitric oxide synthase (iNOS) is stimulated by inflammation in epithelial cells. Therefore, the upregulation of NO by iNOS under inflammation indicates that NO may serve as an informative biomarker of depression.

There are two major regulators, NorR (nitric oxide sensing regulator) and NsrR (nitric oxide-sensitive transcriptional repressor), that mediate the bacterial response to NO stress (Spiro, 2007; Stern and Zhu, 2014). NorR directly binds NO and regulates genes for NO detoxification in *E. coli* and *V. cholerae* (Figure 2Aiii) (D'Autréaux et al., 2005; Stern et al., 2012). To leverage these natural NO-sensing parts, NorR was coupled to the regulation of a DNA recombinase, which allows for the permanent recording of NO signals associated with gut inflammation tested in explant cultures of mouse ileum (Archer et al., 2012). For optimizing NO biosensors, a positive feedback loop was introduced to fine-tune the level of NorR in *E. coli* Nissle 1917 (Chen et al., 2021). With a positive feedback loop, the NO also induces the NorR expression, and the increase of activated NorR provides the biosensor with a larger dynamic range.

The reporting of symptoms related to inflammation can vary from person to person, which may complicate the clinical correlation of digestive inflammation with a neurological disorder. The development of NO and tetrathionate biosensors offers new tools to correlate the earliest stages of inflammation with depression-associated gut-brain axis dysregulation.

### Biosensors for General Gut Signals

The spatial heterogeneity of microbial populations across the digestive tract is challenging to detect in live organisms (Donaldson et al., 2016). This spatial heterogeneity is rooted in the variation of metabolite producers, metabolite sinks, and diffusion from the production site to the detection site. This requires approaches to resolve metabolite spatial heterogeneity. The mapping of signals across the digestive tract requires landmark signals that can report on the address of detected signals. Two parameters that vary throughout the digestive tract tissues are oxygen (O_2_) and pH (Carreau et al., 2011; Aoi and Marunaka, 2014; De Santis and Singer, 2015). Oxygen concentration decreases from tissue to lumen. Also, the small intestine has a higher O_2_ level and lower pH, while the large intestine has a lower O_2_ level and higher pH. Coupling O_2_ sensing (Figure 2Aiv) and pH sensing (Figure 2Av) with the ability to sense biomarker metabolites would enable the spatiotemporal sensing capabilities needed for engineered bacteria to target diseases precisely.

Recently, work from the Tal Danino group engineered bacteria to carry a AND circuit of hypoxia and lactate sensors (Chien et al., 2021). The AND gate is built based on two transcriptional regulators. The fumarate and nitrate reduction regulatory protein (FNR) senses O_2_ level (Figure 2Aiv). FNR was engineered to regulate *asd*, an essential gene that encodes an aspartate-semialdehyde dehydrogenase for the lysine, threonine, and methionine biosynthesis. In tandem, the *l* -lactate dehydrogenase operon regulatory protein (LldR) senses lactic acid. LldR was engineered to regulate *glms*, another essential gene that encodes a glucosamine-6-phosphate synthase for the biosynthesis of building blocks for the bacterial cell wall. Therefore, bacterial replication is only permitted when both essential genes are expressed when the environmental O_2_ and lactic acid are in the designated range for the *S. Typhimurium* ELH1301 *Δasd Δglms*. *S. Typhimurium* was used in cancer therapy because its population increases and accumulates around tumors. However, *S. Typhimurium* can also localize to the liver and spleen (Hoffman, 2016). Because hypoxia and high lactate are unique signals in tumor environments, in mice experiments, these hypoxia-lactate sensing *S. Typhimurium* will improve safety with increased tumor specificity compared to the O_2_-only and lactate-only sensing strains. Similar genetic circuits might be applied for identified tissue microenvironments in which the O_2_ levels and pH are dramatically different (Pereira and Berry, 2017; Singhal and Shah, 2020). When sensing O_2_ and pH coupled with depression biomarker sensors, biosensors with complex circuits may identify specific microbial niches that produce depression-associated biomarkers.

## Output for Biosensors

### Strategies to Map the Signaling Landscape Using FRET

Microbial communities can be spatially and compositionally heterogeneous and change with maturation. One strategy discussed above is the regulation of bacterial growth to defined niches. Based on signal sensing, outputs can control the expression of essential genes like *asd* and *glms* discussed above (Figure 2Bi). A second approach applies imaging technologies to map how biomarker signals change with time. Such approaches may have utility in the preclinical studies to connect gut-brain axis dysregulation with depression symptoms. The split fluorescent proteins (Figure 2Bii) and FRET (Figure 2Biii) have been developed as detecting approaches. The binding of signaling molecules to the FRET biosensor results in conformational changes that alter the distance between the donor and acceptor fluorophores leading to changes in the FRET signal.

In the I3AA FRET biosensor we discussed above (Herud-Sikimić et al., 2021), the binding of I3AA to the repressor induces structural changes that can be detected by FRET. A similar FRET strategy was applied to histidine kinase, the protein that senses environmental signals in the bacterial TCS (Duvall and Childers, 2020). In this example, the *Caulobacter crescentus* kinase CckA was engineered to contain both donor and acceptor fluorescent proteins. The authors demonstrated that the engineered histidine kinase successfully responds to cyclic-di-GMP. This FRET-based approach could potentially be applied to the I3A-sensing (Wang et al., 2021) and the tetrathionate-sensing histidine kinases (Daeffler et al., 2017) to spatially and temporally map these signals within microbial populations.

A third strategy has utilized circular permuted GFP, whose fluorescence is sensitive to ligand binding. This strategy has been recently applied to engineer a sensor for GABA (*γ*-aminobutyric acid) (Marvin et al., 2019), a major inhibitory neurotransmitter of the central nervous system (CNS). The biosensor design included inserting a circularly permuted fluorescent protein into the *Pseudomonas fluorescens* GABA binding protein Pf622. This GABA biosensor was applied to imaging experiments in mice and zebrafish. Bacterial gut commensals have also been found to have the enzymatic capacity to decarboxylate glutamate to generate GABA (Pokusaeva et al., 2017; Strandwitz et al., 2019). Therefore, future development of GABA biosensors applied towards microbial communities will be insightful.

### Acoustic Gene Reporters (AGRs) Enable Detection of Biomarkers by Ultrasound

Along the longitudinal and transverse axes in the gut, distinct habitats create a heterogeneous distribution of microbes and their metabolites (Tropini et al., 2017). While engineered biosensors would enable spatiotemporal sensing capabilities, visualization of bacteria in the gut will be required for resolving spatial heterogeneity of microbial populations. Fluorescence imaging approaches are limited in application as light cannot penetrate deeply into tissues. Ultrasound detection methods have been developed to track signals deep within tissues (Figure 2Biv). These technologies leverage gas vesicles (GVs), protein nanostructures found from diverse bacteria and archaea (Walsby, 1994; Pfeifer, 2012). GVs are filled with gas and thus contribute to buoyancy. The unique features of GVs allow for the scattering of sound waves (Shapiro et al., 2014). Therefore, GVs present opportunities for detecting signals by ultrasound, which allow deep penetrant imaging with a <100 µM spatial resolution. This technology has been developed with engineered GV genes as acoustic reporter genes (ARG). Furthermore, their expression in bacteria and detection by ultrasound have been demonstrated (Bourdeau et al., 2018).

Genetic engineering has tuned the sensitivity of GVs to varied acoustic pressure (Figure 2Biv) (Shapiro et al., 2014). Therefore, distinct GVs can be distinguished by their sensitivity to acoustic pressure (Lakshmanan et al., 2016; Hurt et al., 2021) to monitor two signals simultaneously. The ARGs have been engineered with improved expression (Lakshmanan et al., 2017; Hurt et al., 2021) and various acoustic properties (Bourdeau et al., 2018; Hurt et al., 2021) in different cell types, such as *E. coli* and *Salmonella typhimurium* (Bourdeau et al., 2018). The engineered bacteria with ARGs were also tested deep inside a mammalian host within the tumors (Hurt et al., 2021) and gastrointestinal tract (Bourdeau et al., 2018). ARGs thus present maturing diagnostic technologies for real-time reporting of gut-brain axis dysregulation.

### Strategies to Record Transient Signals as Long-Term Memories

While FRET biosensors offer real-time monitoring, the longevity of these signals is limited by the protein’s half-life, which can span over the time frame of ∼30 min to 20–200 h (Eden et al., 2011; Schwanhäusser et al., 2011). Longer-term storage of signals has utilized recombinases, enzymes that invert the orientation of a piece of DNA (Figure 2Bv). The recombinases-based genetic circuit allows recording in the form of altered DNA upon transient exposure to signal. The recorded information in DNA can be recalled later by PCR, which allows the engineered biosensor to maintain a long-term memory (Yang et al., 2014).

As an alternative, the toggle switch-like circuit can also record and store signals for a long time (Figure 2Bvi). A toggle switch is a bistable gene-regulatory network (Gardner et al., 2000). The two repressors mutually repress the expression of the other in a toggle switch, which flips between the two states. In a biosensor, the expression of one repressor is induced by the signal. The induction of one repressor turns and keeps the toggle switch into one of the states. Thus, the biosensor will sense and memorize the signal and can be read out through the analysis of samples such as stools.

## Discussion

While good initial progress has been made in identifying biosensor input and output strategies, several steps will be needed to apply these biosensors to study mental health. This includes validating and tuning current biosensors for metabolite surveillance within the mouse and human gut microbiome environments. For example, studies will be needed to quantify and compare biomarker signal levels under health and disease conditions, and this may require engineering biosensors’ IC_50_ for needed sensitivity. Beyond measurement validation, the metabolic landscape within the mouse gut contains multiple sources and sinks. The flux of gut metabolites may be variably directed towards other non-depression-associated pathways. Therefore, preclinical studies in mice correlating biosensor readout with depression symptoms and metabolite levels in the brain will be needed.

Moreover, while FDA approved the first cell therapy in 2017 (Braendstrup et al., 2020), the and microbial sensors have been tested in a pig, biosensors remain to be deployed in humans (Mimee et al., 2018). Regarding the use of microbial biosensors, ensuring biosafety is another challenge. To this end, bacterial pathogenicity, survival, and the risk of mutation should be minimized and under strict control (McNerney et al., 2021).

### Need for Biomarkers Landscapes to Specifically Diagnose Diseases in Mental Health

Another major challenge for cell-based diagnostics and therapeutics is how to target diseases of interest specifically (Cubillos-Ruiz et al., 2021). Currently known signals are associated with a wide range of diseases for the microbial biosensors discussed above. For example, indole(s) and indole-producing bacteria are correlated with inflammatory bowel diseases (IBD), metabolic diseases, neurodegenerative diseases, and cancers (Lee et al., 2015). In addition, many mental health diseases share overlapping symptoms reported by patients (Ronald et al., 2008; Grisanzio et al., 2018; Plana-Ripoll et al., 2019), which present challenges in the deconvolution of many diseases correlated with variations in the microbiome. This suggests a need for additional biomarkers with improved specificity to distinguish depression from other mental health disorders. Alternatively, with only low to moderate specificity biomarkers, strategies to detect a suite of biomarkers may provide another route to increase specificity.

As an example of integrating multiple cues, hypoxia-lactate sensing *S. Typhimurium* has increased tumor specificity (Chien et al., 2021). Recent work demonstrated that living cells that sense up to three signals precisely recognize targeted cancer (Williams et al., 2020). New studies will be needed to demonstrate how integrated sensing of multiple cues correlates with mental health disease states. Towards the goal of signal integration, there has been success in both protein-based (Ho et al., 2021; Vishweshwaraiah et al., 2021) and transcriptional logic gates to enhance specificity by processing multiple inputs (Cubillos-Ruiz et al., 2021).

In bacteria, TCSs exhibit diverse mechanisms to integrate and process multiple signals. TCSs process multi-inputs through kinase networks. Crosstalks between multiple kinases increase the signal integration capabilities of those networks (Francis and Porter, 2019). In addition, kinase signaling pathways integrate signals through tandems sensors where a single histidine kinase has more than one sensory domain. Interestingly, natural (Fang et al., 2015; Mann et al., 2016) and synthetically engineered (Moglich et al., 2010) tandem sensors were found integrating signals as logic gates. The design principles for how tandem sensor structure encodes logic remain poorly developed. We envision that the rapid development in machine learning-based protein structure prediction (Baek et al., 2021; Tunyasuvunakool et al., 2021) and engineering (Yang et al., 2019) will increase our capabilities to design and understand the mechanisms of tandem sensor processing.

### Need for Biosensors of Other Known Biomarkers in the Gut

While progress has been made towards biosensors for indole metabolites, inflammation, pH, and oxygen, new biosensors for many biomarkers of mental health issues will be needed. Notably, we currently lack well-characterized microbial biosensors for kynurenine metabolites. Another key family of depression-associated metabolites is short-chain fatty acids (SCFAs). By fermentation of unabsorbed foods within our digestive tract, the microbiota produces SCFAs (Dalile et al., 2019) that include acetate, butyrate, and propionate (Erny et al., 2015; Kelly et al., 2019). These SCFAs impact the central nervous system through orphan G protein-coupled receptors (GPCRs) (Dalile et al., 2019) and histone deacetylases (HDACs), which impact depression and other mental diseases (Waldecker et al., 2008; Soliman and Rosenberger, 2011). These results suggest the need to develop bacterial biosensors for SCFAs as biomarkers of gut-brain axis dysregulation.

The characterization of the I3A and tetrathionate sensing histidine kinases highlights the potential sensing capabilities of bacterial TCS. We hypothesize that microbes colonized in the gut provide an extensive treasure trove of sensory domains. Furthermore, gut microbes constantly respond to metabolites fluctuation at the colonized location. Therefore, characterization of poorly studied TCSs in the genome of gut microbes may reveal new sensing interchangeable parts to construct biosensors.
